# Hybrid Ablation for Patients with Persistent and Long-Standing Persistent Atrial Fibrillation

**DOI:** 10.3390/jcdd13100505

**Published:** 2026-10-08

**Authors:** Ji-Cheng Hsieh, James K. Gabriels, Kristie Coleman, Stuart Beldner, Frank Manetta, Stavros E. Mountantonakis

**Affiliations:** Cardiovascular Institute, North Shore University Hospital, Northwell, New Hyde Park, NY 11042, USA

**Keywords:** persistent atrial fibrillation, long-standing persistent atrial fibrillation, epicardial–endocardial dissociation, catheter ablation, surgical ablation, hybrid ablation

## Abstract

Atrial fibrillation (AF) is the most commonly encountered arrhythmia, with a rapidly growing prevalence and significant cost to the global healthcare system. Left untreated, AF progresses over time from paroxysmal AF to persistent AF (perAF) and long-standing persistent AF (LS-perAF). AF is associated with significant mortality and morbidity by increasing the risk of developing cardiomyopathy, heart failure, dementia, stroke and systemic embolization. Endocardial catheter ablation (CA) targeting pulmonary vein isolation (PVI) alone is the most commonly employed treatment strategy for patients undergoing invasive treatment for all forms of AF. Despite technological improvements such as pulsed field ablation (PFA), procedural success rates in preventing AF recurrences in patients with perAF and LS-perAF remain suboptimal. A lack of lesion transmurality gives rise to epicardial–endocardial dissociation, which may contribute to AF recurrences. Hybrid ablation (HA) combines epicardial surgical ablation with endocardial CA. HA leads to greater freedom from AF in advanced AF compared to CA, and while existing data support the safety of HA, HA inherently is more invasive, requires a multidisciplinary approach, and has a 5–7.8% risk of major adverse events. HA is reasonable in patients with symptomatic, drug-refractory perAF or LS-perAF. Future directions include evaluating the role of novel operative technologies in HA.

## 1. Introduction

Atrial fibrillation (AF) is the most commonly encountered arrhythmia worldwide, with an estimated prevalence between 2019 and 2023 of 4.8 million cases in the United States alone [1]. AF typically begins as paroxysmal AF, with episodes terminating within seven days. Over time, AF progresses to persistent AF (perAF), with episodes lasting greater than seven days, and long-standing persistent AF (LS-perAF), which is defined as AF that continues for greater than one year [2,3]. Progression of AF from paroxysmal to perAF within 12 months occurs in up to 15% of patients [4]. Proposed mechanisms causing AF include automatic foci, reentrant wavelets, and reentrant circuits [5]. Both triggers and an arrhythmogenic substrate are necessary for AF, and as AF progresses, atrial remodeling leads to perAF and LS-perAF, which are maintained by a substrate as opposed to paroxysmal AF, which is typically driven by triggers [5].

AF is associated with 1.5–2-fold increase in mortality and increased risk of cardiomyopathy, sudden cardiac death, heart failure, vascular dementia, stroke and systemic embolization [2,6,7]. Even in patients without overt clinical stroke, AF is associated with greater decline in cognitive testing performance and reduced brain volume [8,9]. AF predisposes patients to an increased risk of cardiovascular hospitalization, with over 460,000 AF-related hospitalizations yearly and a total annual cost of $26 billion on the United States healthcare system [10]. With significant mortality and morbidity associated with AF, it remains imperative to effectively treat AF and durably restore sinus rhythm.

In this narrative review, we outline ablative treatment options for patients with AF. Our review focuses on hybrid ablation (HA), an established treatment option for patients with perAF and LS-perAF. A literature search was conducted using the PubMed database and articles were identified and selected based on relevance to the scope of the review. While there may be the possibility of incomplete coverage or selection bias, our review aims to provide an introductory review of HA.

## 2. Types of Ablation for Atrial Fibrillation

### 2.1. Endocardial Catheter Ablation

Ablation strategies to treat AF are broadly categorized into endocardial catheter ablation (CA) and epicardial surgical ablation (SA). CA utilizes a minimally invasive transseptal approach with a catheter-based energy source to apply lesions to the endocardial surface of the left atrium with the goal of isolating the pulmonary veins (PVs) [5]. Additional ablation can be performed to target non-PV triggers and an arrhythmogenic substrate [5]. Energy sources are classified as thermal ablation, which includes radiofrequency (RF) ablation, cryoballoon (CB) ablation and laser balloon ablation, or nonthermal ablation, such as pulsed field ablation (PFA) [5]. Unipolar or bipolar RF ablation utilizes an electrode catheter to heat tissue, which results in myocardial scarring [5]. CB ablation utilizes a balloon filled with refrigerant to freeze the myocardium [5]. Laser balloon ablation employs a fiber optic endoscope that produces light energy [5]. PFA is a novel technology that utilizes irreversible electroporation (IRE) [11]. IRE is based on the application of electrical energy to cardiac myocytes [11]. This triggers hyperpermeability of the cell membrane, which leads to cellular death without thermal injury to surrounding tissues and anatomic structures [11].

An early rhythm control strategy of AF with antiarrhythmic drugs (AADs) or endocardial CA is associated with a lower risk of cardiovascular death and stroke [12]. Pulmonary vein isolation (PVI), which is the cornerstone of CA, utilizes circumferential lesions around the right and left PVs to electrically isolate PV triggers that initiate AF [5,13].

### 2.2. Epicardial Surgical Ablation

SA may be considered as a first-line therapy in patients with AF who are undergoing cardiac surgery or a second-line therapy in patients with symptomatic AF who have failed a trial of AADs [3,5]. The MAZE procedure was the earliest surgical approach to rhythm control, applying a “cut and sew” technique to create a biatrial lesion set made of suture lines [14,15]. The MAZE procedure necessitates cardiopulmonary bypass and median sternotomy, and so was often performed concomitantly with cardiac surgery [15,16]. The MAZE procedure was frequently complicated by sinus node dysfunction requiring permanent pacemaker implantation, which limited its adoption and prompted development of alternative techniques [16].

SA has shifted towards minimally invasive approaches such as a mini-thoracotomy approach, a video-assisted thoracoscopic approach, or a robotic-assisted thoracoscopic approach, utilizing bipolar and unipolar RF, microwave, or cryoenergy to create a lesion set mirroring the suture lines of the original MAZE procedure [5,15,17,18,19]. For example, the Cox-Maze IV procedure incorporates cryoablation and RF ablation [19]. Bipolar RF ablation is preferred over unipolar RF ablation due to a greater ease in creating transmural lesions.

## 3. Limitations of Endocardial Ablation

### 3.1. Outcomes Following Pulmonary Vein Isolation

Guidelines recommend PVI as the primary lesion set of choice for patients undergoing CA [3]. PVI is more efficacious with respect to reducing AF recurrences when compared to AADs [5,20]. CA is recommended as a first-line therapy in the management of paroxysmal or perAF [5]. Success rates for CA in patients with paroxysmal AF range from 59 to 89% freedom from AF at 12 months and 45 to 63% freedom from AF at 24 months [5]. For patients with perAF, success rates range from 61 to 74% freedom from AF by 12 months and reach 54% freedom from AF at 24 months [5,21].

A 2017 systematic review and meta-analysis reported 66.7% arrhythmia-free survival without AADs following PVI in patients with perAF at 12 months [22]. Freedom from AF without AADs, as measured via ECG and a 24 h Holter monitor, in patients with perAF and LS-perAF, decreases to 43% at 2 years and 20% at 5 years [23,24]. By comparison, freedom from AF without AADs, as measured via ECG and a 24 h Holter monitor in patients with paroxysmal AF, ranges from 46.6 to 71% at 5 years [25,26]. When comparing the success rates between various energy modalities, RF and CB ablation are equally efficacious, and despite shorter procedural times with PFA, procedural success rates remain similar [27,28,29]. The SPHERE PER-AF trial found that a dual-energy RF/PFA ablation catheter was noninferior to traditional RF ablation in patients with perAF [30]. The PULSED-AF trial reported a 55.1% freedom from AF without AAD adjustment following PVI performed using PFA at 12 months in patients with perAF [31]. Despite technological advancements in endocardial CA using PFA, procedural success rates still remain suboptimal.

Predictors of ablation failure include greater left atrial size, age greater than 70 years, duration of perAF, and total AF duration [23,24,26,32]. Patients with a total AF duration > 2 years have three times the risk of AF recurrence when compared to patients with AF duration < 2 years [24]. Recurrence of AF following PVI is most commonly due to a lack of lesion transmurality and PV reconnections [5]. With multiple subsequent ablations, freedom from AF off AADs in perAF and LS-perAF increases to 69% at 2 years and 45% at 5 years [23,24].

Procedure-related complications of CA using RF and CB include stroke (<1%), cardiac tamponade (1–2%), phrenic nerve injury (1–2%), PV stenosis (<1%) and atrioesophageal fistula (AEF) (0.02–0.11%) [11,33]. Although the incidence of an AEF is low, this complication is often fatal [34]. Current data support the notion that complications of PFA, such as permanent phrenic paralysis, AEF and PV stenosis, have not yet been reported in the literature. Hemolysis resulting in the need for renal replacement therapy and coronary artery vasospasm are known complications from PFA [35].

### 3.2. Outcomes of Lesion Sets Beyond Pulmonary Vein Isolation

The efficacy of alternative lesion sets performed in conjunction with PVI has been assessed with the goal of identifying additional treatment strategies to improve outcomes in patients with perAF and LS-perAF. Additional lesion sets serve to treat non-PV triggers, treat inducible AFL/AT and perform substrate-based ablation [5]. These lesion sets include posterior wall isolation (PWI), mitral isthmus lines, superior vena cava isolation, left atrial appendage (LAA) isolation, cavotricuspid isthmus (CTI) ablation, focal ablation targeting non-PV triggers, and treatment of complex fractionated atrial electrograms (CFAEs) [5,36,37,38,39]. Recent studies define additional procedures as “PVI-plus,” which encompasses PVI with the addition of linear ablation or ablation of CFAEs [40]. Guidelines recommend against routine addition of lesion sets beyond PVI, as additional ablation has not uniformly been demonstrated to reduce the risk of AF recurrence [3]. Additional lesion sets, however, may be considered if additional triggers of AF are identified [3].

Despite the guideline recommendations, linear lesion sets and ablation of CFAEs are often employed in addition to PVI [5]. Examples of linear lesions include a left atrial “roof” line connecting circumferential left and right PV lesions and mitral isthmus lines [5]. A CTI line may be applied in the setting of concomitant typical atrial flutter (AFL). Care must be taken to ensure that linear lesions are continuous, durable and without gaps [5]. Even when PFA or combined RF and PFA are used to create linear lesion sets, long-term durability remains suboptimal [41,42]. CFAEs are low-voltage electrograms with a high degree of fractionation that can serve as a substrate for AF [5]. CFAEs can be focally ablated, but the utility of ablation is limited when multiple CFAEs are present [5]. STAR AF II found no difference between groups treated with PVI plus linear lesions and PVI plus CFAE ablation in single-procedure freedom from AF with or without AADs at 18 months, as measured using an ECG and 24 h Holter monitor, in patients with perAF undergoing CA [43]. CHASE-AF also demonstrated no differences in freedom from AF/AFL or atrial tachycardia (AT) measured using a 72 h Holter monitor at 12 months in patients with perAF who were treated with PVI alone compared to PVI plus linear lesions and CFAE ablation [44]. Compared to PVI alone, the combination of PVI, CFAE ablation, and linear lesions was found to have the poorest freedom from AF at 24 months, and PVI-plus did not improve arrhythmia-free survival measured with a 24 h Holter monitor at 24 months [23,40]. PVI plus linear lesions and CFAE ablation subjected patients to increased procedure and fluoroscopy times and a greater risk of post-procedural pericarditis [44].

Left atrial PWI is of particular interest due to its shared embryologic origin with the PVs and its arrhythmogenic potential [45]. The CAPLA and POBI-AF trials demonstrated no difference in the rate of AF recurrence at 12 months and 16 months, respectively, between PVI alone and PVI plus PWI in patients with perAF [37,46]. Posterior wall (PW) reconnection via the left atrial roof is common in patients who have undergone PWI using RF ablation [47]. The addition of PWI prolongs procedural and ablation time, and given anatomical proximity to the esophagus, PWI using RF ablation or CB ablation subjects patients to the risk of esophageal injury and AEF [5,46,48,49].

There is a renewed interest in PWI using PFA due to lower risk of esophageal injury with PFA [50]. PFA is both safe and efficacious for PWI, with no reported instances of esophageal ulcerations, dysmotility, or AEF [11,35,45]. In the ADVANTAGE-AF trial, PVI + PWI with PFA in perAF had a 73.4% freedom from AF/AFL/AT off a new or increased dose of AADs, as measured on an implantable cardiac monitor, at 12 months [51]. Outcomes between different lesion sets using PFA remain similar, however, as a retrospective study involving PFA with PVI and PWI compared to PVI alone revealed no significant difference in freedom from AF/AFL/AT at 12 months in patients with perAF [50].

The Vein of Marshall (VOM) is an embryologic remnant of the left superior vena cava [52]. The VOM may be a trigger of AF and additionally contains parasympathetic and sympathetic fibers that drive the progression of AF [52]. The VENUS trial reported a significantly greater freedom from AF/AT without AADs, as measured via a 1-month monitoring device, in patients who underwent concomitant RF ablation and venous ethanol infusion in the VOM compared to CA alone [52]. RF ablation included PVI with the addition of PWI, CTI ablation, and CFAE ablation per operator discretion [52]. Although 16% of patients in the VOM and CA group had unsuccessful cannulation of the VOM, the intention-to-treat analysis including these patients still noted greater freedom with VOM and CA (49.2%) compared to CA alone (38.0%) at 6 and 12 months [52,53]. The Marshall-Plan study found that 86.4% in patients undergoing CA and VOM alcohol ablation had freedom from AF/AFL/AT, as measured via weekly 30 s rhythm strip transmissions, at 12 months, regardless of AAD use [53]. VOM ethanol infusion was successful in 97% of cases [53]. This was a significantly better outcome than in the group of patients who underwent CA alone with PVI (66.1% freedom from AF/AFL/AT, *p* = 0.012) [52,53]. It is important to note, however, that the CA and VOM group utilized a three-step ablation strategy which incorporated VOM ethanol infusion, PVI, and linear roof, mitral, and CTI lines [53].

### 3.3. Endocardial–Epicardial Dissociation

Lesion non-transmurality is an important contributor to recurrence of AF [54]. It was believed that the thin-walled atria inherently would exhibit epicardial–endocardial homogeneity of atrial substrate [54]. However, simultaneous epicardial and endocardial mapping of the left atrial PW in patients with perAF revealed notable differences—higher bipolar voltage and atrial activation in the epicardium when compared to the endocardium, with epicardial to endocardial conduction block [55,56]. In patients undergoing epicardial–endocardial mapping in sinus rhythm as opposed to AF, there were no significant differences in voltage and atrial activation [55]. These findings highlight the importance of the epicardium in potentially sustaining AF and the misconception that endocardial mapping post-ablation will reflect both epicardial and endocardial activity [55]. Endocardial–epicardial mapping of patients undergoing repeat ablation frequently demonstrates electrical dissociation [54,56,57]. This dissociation likely contributes to procedural failure, particularly in perAF and LS-perAF [55].

**Figure 1:**
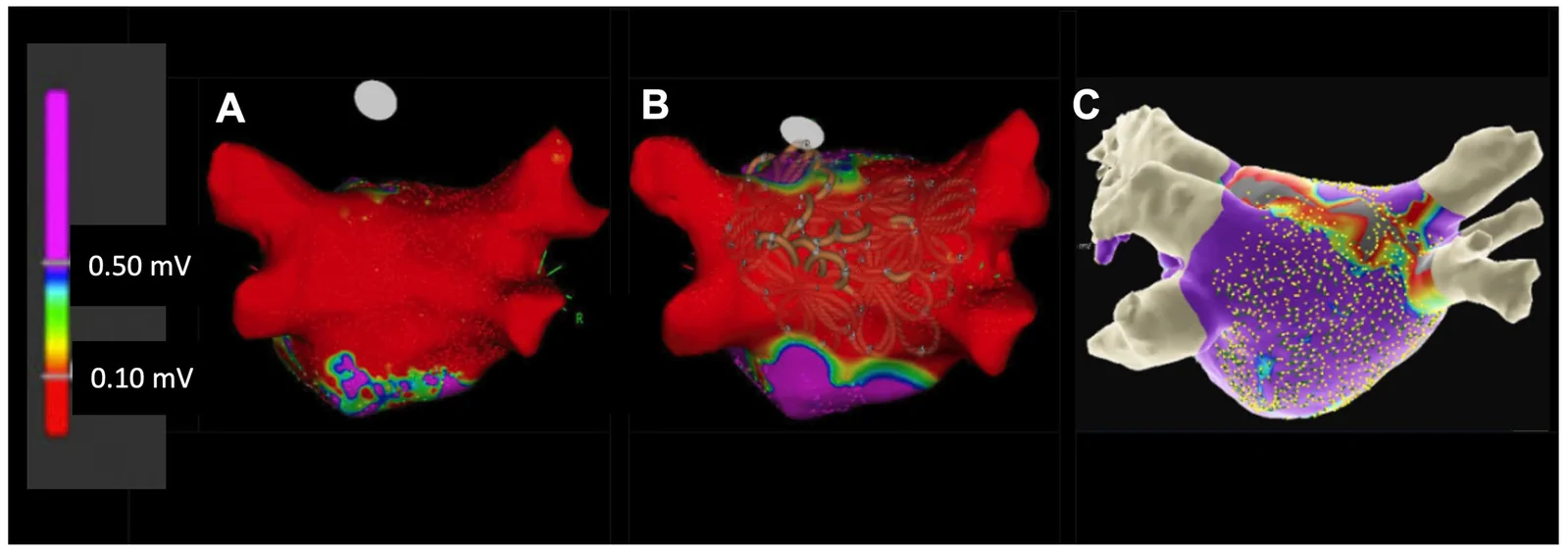
Endocardial–epicardial dissociation following combined endocardial RF ablation and PFA. (**A**) Left atrial endocardial voltage map performed five years after endocardial RF ablation. (**B**) Endocardial bipolar voltage following subsequent PFA of the PW using a pentaspline catheter. (**C**) Epicardial bipolar voltage performed six months after the endocardial PW ablation using PFA. PW: posterior wall, RF: radiofrequency, PFA: pulsed field ablation. Scale for the bipolar maps: 0.5 mV. Adapted with permission from Elkhechen et al. [60].

When RF ablation is used, ensuring transmurality is dependent on RF power and duration, along with catheter stability and contact force [5]. Power and duration are limited by risk of damage to nearby tissue [5]. Comparisons between PFA and RF ablation in patients who have undergone PWI show no significant differences in lesion transmurality in patients with perAF on follow-up epicardial mapping during subsequent HA [58,59]. Figure 1 demonstrates a patient who had undergone PWI with RF ablation five years prior [60]. The patient subsequently underwent a second ablation in which PFA was performed on the PW using a pentaspline catheter despite a lack of endocardial bipolar voltage on the PW [60]. At the time of an epicardial ablation performed six months following the endocardial PW “re-isolation” using PFA, endocardial–epicardial dissociation was evident with persistent epicardial bipolar voltage [60]. It is important to note, however, that while AF recurrence may be explained by endocardial–epicardial dissociation, the presence of such dissociation may not necessarily portend treatment failure or worse clinical outcomes, emphasizing the need for an individualized approach to mapping and ablation.

## 4. Hybrid Ablation

### 4.1. Operative Technique

Hybrid convergent ablation (HA) is a procedure encompassing both epicardial and endocardial ablation by a cardiac surgeon and electrophysiologist. The initial surgical approach for epicardial ablation in HA, as specified in the CONVERGE trial, utilized pericardioscopy with a transdiaphragmatic or subxiphoid approach [61]. An initial transesophageal echocardiogram (TEE) is commonly performed to assess the LAA anatomy and confirm the absence of intracardiac thrombi [62]. With this approach, epicardial PWI was performed with the AtriCure EPi-Sense System, a unipolar RF catheter FDA approved for use in HA [61]. In the CONVERGE trial, epicardial mapping was not performed. Subsequent endocardial CA utilized RF to complete PVI and included a CTI line in the lesion set [61]. Endocardial mapping and touch-up RF ablation of lesion gaps was then performed, followed by standard confirmation of an entrance and exit block to assure PVI [61].

Subsequent trials, most notably CEASE-AF and HARTCAP-AF, utilized a unilateral or bilateral video-assisted thoracoscopic approach for the initial epicardial ablation using the AtriCure Synergy System and a bipolar RF clamp [63,64]. With the AtriCure Synergy System, PVI and PWI are both performed epicardially [62,64]. A bilateral video-assisted thoracoscopic approach was chosen if a left-sided unilateral approach did not appropriately access the right PVs [62]. This initial procedure also included management of the LAA with clipping or a closure device [62,64]. Standard endocardial mapping, touch-up RF ablation, and confirmation of entrance and exit block were then performed to complete the procedure [62,64].

**Figure 2:**
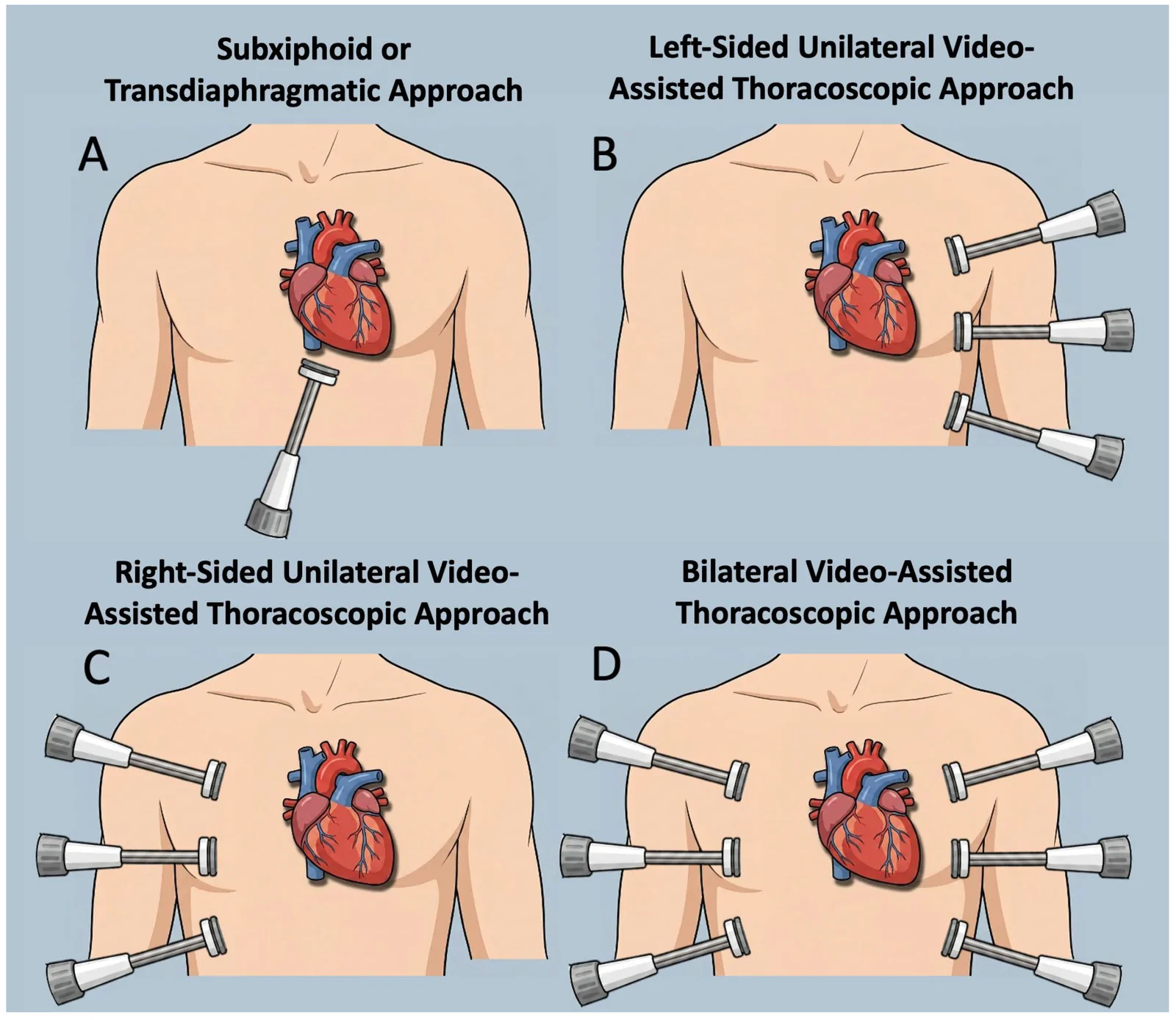
Surgical approaches for epicardial access during a HA. (**A**) Subxiphoid or transdiaphragmatic approach. (**B**) Left-sided unilateral video-assisted thoracoscopic approach with ports in the third, fifth, and seventh intercostal spaces. (**C**) Right-sided unilateral video-assisted thoracoscopic approach. (**D**) Bilateral video-assisted thoracoscopic approach with ports in the third, fifth, and seventh intercostal spaces. HA: hybrid ablation.

**Figure 3:**
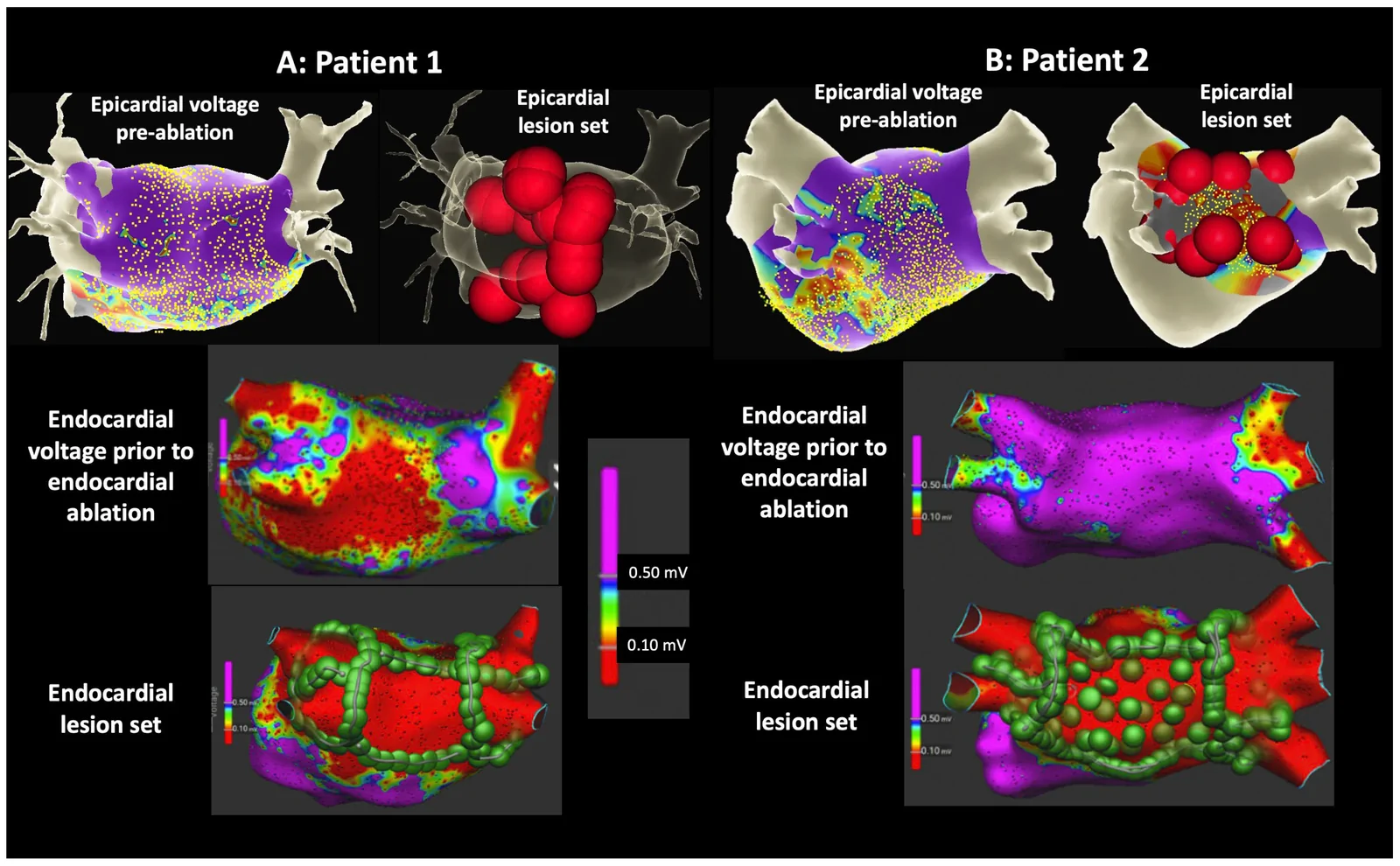
Endocardial–epicardial dissociation following epicardial RF ablation in patients with LS-perAF undergoing de novo HA. Posterior–anterior views of voltage maps from two patients who underwent HA. (**A**) Patient 1 had LS-perAF (five years in duration). She underwent epicardial mapping and ablation using a unilateral left-sided robotic-assisted video-assisted thoracoscopic surgical approach. The epicardial lesion set is shown (red spheres correspond to lesion locations). Thirteen months following her epicardial ablation she returned for the endocardial portion of her HA. Prior to endocardial ablation, her PW demonstrated low bipolar voltage corresponding to the location of the epicardial lesions (note the normal voltage on the PW near the right PVs—due to incomplete epicardial ablation from the left-sided approach). The endocardial lesion set is shown (PVI and PWI); green spheres correspond to locations of PFA lesions. (**B**) Patient 2 had long-standing persistent AF (LS-perAF) (one year in duration). He underwent epicardial mapping and ablation using a unilateral left-sided robotic-assisted video-assisted thoracoscopic surgical approach. The epicardial lesion set is shown (red spheres correspond to lesion locations). At the time of his endocardial ablation performed five months later, there was no evidence of transmurality from the epicardial ablation. Endocardial roof and floor lines, along with homogenization of the PW, were performed along with PVI. HA: hybrid ablation; LS-perAF: long-standing persistent atrial fibrillation; PV: pulmonary vein; PVI: pulmonary vein isolation, PW: posterior wall; PWI: posterior wall isolation; PFA: pulsed field ablation. Original figure, not published elsewhere.

An emerging novel technique in HA is a robotic-assisted thoracoscopic approach for the initial SA [63]. A robotic approach with the Da Vinci Xi System (Intuitive Surgical Operations) offers 3-D visualization, magnification, and greater control, which allows for reduced operative time and safer dissection of and access to key anatomic structures [18,63]. There is also growing interest in epicardial electroanatomic mapping during the epicardial portion of the procedure using a high-density mapping catheter. Intraoperative epicardial electroanatomic mapping can be used to confirm uniform epicardial electrical isolation [63,65]. Similar to endocardial electroanatomic mapping, an epicardial low voltage is not sufficient to demonstrate lesion transmurality. Figure 2 depicts the different surgical approaches utilized for SA in patients undergoing HA. Following epicardial ablation, endocardial electroanatomic mapping can reveal the presence or absence of transmural ablation, again demonstrating endocardial–epicardial dissociation (Figure 3).

Epicardial and endocardial ablation may be performed in series within a single procedure or as a staged procedure [61,62,64]. A patient may be managed more efficiently with a single procedure, as a staged approach with a 6–18-week interval will expose the patient to a second admission and greater procedural risk [64]. The greatest benefit of a staged approach, however, is the interval post-operative maturing of epicardial lesion sets and resolution of tissue edema and pericardial inflammation, which allows for more accurate subsequent endocardial mapping and touch-up ablation [64]. Staging the procedure also obviates the need for therapeutic anticoagulation in a patient immediately following cardiac surgery.

As for follow-up after HA, the initial 3-month blanking period after ablation is deferred due to frequent arrhythmia recurrence, which does not suggest long-term ablation failure [5]. Post-operative rhythm follow-up after HA typically occurs at 3, 6, and 12 months in the first year, with annual follow-up thereafter [61,62,64]. A 12-lead ECG and a 24 h Holter monitor can be used during 3- and 6-month follow-up, while annual follow-ups commonly use a 7-day wearable monitor [61,62,64]. Measures of success vary from procedural success such as confirmation of PVI, reduction in AF burden, or freedom from AF/AFL/AT lasting longer than 30 s [5]. As the goal of ablation is ideally to eliminate AF, freedom from AF/AFL/AT is commonly reported [5].

### 4.2. Hybrid Ablation Outcomes

The CONVERGE, CEASE-AF, and HARTCAP-AF trials were large prospective randomized controlled trials that assessed clinical outcomes of a hybrid convergent approach compared to RF CA in patients with perAF and LS-perAF with no history of prior ablations [61,62,64]. While all trials involved a combined approach with SA and CA, the operative technique and approach varied. CONVERGE utilized a single-procedure HA with SA and CA performed in series, with initial SA using a unipolar catheter and a transdiaphragmatic or subxiphoid approach [61]. CEASE-AF utilized a staged HA procedure with an index SA using a bipolar RF clamp and a bilateral video-assisted thoracoscopic approach followed by CA 91–180 days later [64]. HARTCAP-AF utilized single-procedure HA with SA and CA performed in series, with initial SA completed with a bipolar clamp and a unilateral or bilateral video-assisted thoracoscopic approach [62]. Study population characteristics and operative approaches are summarized in Table 1.

Greater freedom from AF/AFL/AT without new or increased doses of AADs at 12 months was noted with HA in CONVERGE (67.7% HA vs. 50.0% CA) and CEASE-AF (71.6% HA vs. 39.2% CA), and greater freedom from AF/AFL/AT off AADs was seen in HARTCAP-AF (89% HA vs. 41% CA) [61,62,64]. This benefit was seen at 18 months in subgroup analysis of patients with LS-perAF in CONVERGE (60.5% in HA vs. 25.9% in CA) [61]. Additionally, significant benefit was also noted at 24 months in CEASE-AF (66.3% in HA vs. 33.3% in CA), and 36 months in HARTCAP-AF (57.0% in HA vs. 22.7% in CA) [62,64]. While these results should be interpreted judiciously given the notable variation between trials with respect to operative technique and energy, they support the use of a combined endocardial and epicardial approach, whether in series or staged, in perAF and LS-perAF [61,62,64,66]. Intertrial comparisons of procedural success are more limited given the aforementioned variation in technique and, more importantly, differences in post-operative rhythm monitoring with 12-lead electrocardiogram, a 1–2-day Holter monitor, or a 7-day wearable cardiac monitor. The primary endpoint outcomes are summarized below in Table 2.

Possible explanations for the benefits of HA include the direct visual confirmation afforded by epicardial SA, which allows for higher quality lesion sets [15]. It is believed that HA is more effective than CA due to greater lesion transmurality and procedural ease in accessing the PW [64]. The nature of the epicardial ablation reduces the risk of injury and allows for a more aggressive ablation strategy [64]. Epicardial ablation of the PW directs energy away from the esophagus [61,64]. The addition of a box lesion to isolate the PW at the time of an epicardial ablation increases freedom from AF on early follow-up [67].

LAA isolation was performed in a majority of patients in the CEASE-AF and HARTCAP-AF trials, where a thoracoscopic approach was used: 92% and 100% of patients received clipping or a closure device, respectively [62,64]. It remains unclear if the addition of LAA isolation in HA reduces AF recurrence; a recent small single-center study reported a trend towards reduction in AF recurrence from 44% to 30% that did not reach significance (*p* = 0.067) [68]. Management of LAA in HA contrasts with other approaches, such as in the AMAZE trial, which utilized endocardial PVI plus a subxiphoid percutaneous approach to LAA ligation [69]. AMAZE found no difference in procedural success with the addition of percutaneous LAA ligation to PVI [69].

Epicardial and endocardial mapping allows for thorough electrophysiologic assessment of the lesion set, appropriate assessment of lesion transmurality, and identification of gaps that can be immediately addressed intraoperatively [15,54]. Endocardial touch-up ablation helps address lesion gaps left behind by initial epicardial ablation (Figure 3). The CONVERGE and HARTCAP-AF trials, in which HA was performed as a single procedure, had 38% and 42% of patients, respectively, receive endocardial touch-up ablation [61,62]. In CEASE-AF, where patients received a staged procedure with subsequent endocardial touch-up ablation up to six months after the initial epicardial ablation, 91% of patients received subsequent endocardial touch-up ablation [64].

While it is difficult to explain this discrepancy in rates of touch-up ablation between CONVERGE and CEASE-AF due to differences in energy (unipolar vs. bipolar) and approach (subxiphoid/transdiaphragmatic vs. unilateral/bilateral video-assisted thoracoscopic), a more appropriate comparison can be made between HARTCAP-AF and CEASE-AF, as both studies utilized bipolar RF clamps, a video-assisted thoracoscopic approach, and a PVI and PWI lesion set with LAA closure or clipping. The key difference between HARTCAP-AF and CEASE-AF is a single-procedure HA in HARTCAP-AF and a staged HA procedure in CEASE-AF, and it is likely that lesion set maturation and resolution of edema and inflammation may have led to more accurate identification of lesion gaps that otherwise would have been identified during a single-procedure HA. How lesion sets mature over time and what factors lead to a lesion gap remains to be investigated.

Periatrial epicardial fat is thought to promote local inflammation and fibrosis, and is associated with the development and persistence of AF and recurrence after CA [70]. Ensuring lesion transmurality may be more difficult with significant epicardial fat, especially with a unipolar RF catheter, but HA utilizing a bipolar RF clamp did not observe any effect of epicardial fat on procedural success [70].

The total procedure time of HA is significantly longer than for CA, but the fluoroscopy and procedural time of the endocardial CA portion in HA tends to be shorter when compared to CA alone [61,64]. Acute procedural success, defined as completion of lesion set and confirmation of conduction block, was not different between HA and CA [61,64]. Post-procedural quality of life was also not different between HA and CA; the reduction in AF burden is thought to balance out the increased post-operative recovery time in HA [62].

### 4.3. Patient Selection

HA is one of many ablation strategies available to electrophysiologists in treating patients with perAF and LS-perAF. Management strategies include CA alone, single-procedure HA with SA and CA in series, or a staged approach with initial SA followed by completion of PVI and touch-up ablation of the PW. In most cases, CA alone is the appropriate choice. CASA-AF found that CA and SA in LS-perAF were similar in outcomes of freedom from AF/AT at 12 months without AADs with respect to a reduction in AF/AT burden, but CA was significantly more cost effective and provided greater quality-adjusted life-years compared to SA [71].

While the inclusion of SA during cardiac surgery has been known to increase the risk of pacemaker placement, in a direct comparison between SA with a thoracoscopic approach and CA in CASA-AF, there was a nonsignificant trend towards a greater 30-day complication rate in the SA group, with one patient death, one patient requiring pacemaker insertion, and two patients with hemi-diaphragmatic paralysis or paresis [3,71]. Patients with AF and comorbidities, who may have an increased operative risk, may be better suited for CA for rhythm management.

Patients with comorbidities associated with higher risk of ablation failure, such as prior failed CA, longer AF duration, planned cardiac surgery, or a contraindication to anticoagulants that warrants surgical management of the left atrial appendage, favor a more aggressive ablation strategy involving SA, such as single-procedure HA or a staged approach, especially in patients with low operative risk. The 2016 ESC guidelines recommend SA as second-line therapy in patients who failed initial CA [33]. The effectiveness of CA decreases with greater AF duration, particularly past 12 months [5]. The 2023 ACC/AHA guidelines report a Class IIb, Level B-R recommendation for hybrid epicardial–endocardial ablation in patients with symptomatic, drug-refractory perAF, and the aforementioned landmark studies provide the strongest evidence in supporting the use of HA in patients with perAF and LS-perAF [3,61,62,64]. In these patients, there is benefit to HA, despite greater procedural risk compared to CA [61].

Given the ease and safety of PWI with SA, and the ability of LAA clipping or device closure, SA with a video-assisted thoracoscopic or robotic-assisted approach is favored for the greatest benefits in patients who are undergoing cardiac surgery or require management of the left atrial appendage. A traditional subxiphoid approach to initial SA in HA, as demonstrated in CONVERGE, can be limited in patients with large body habitus, due to difficulty in advancing the pericardioscopic cannula and ablation catheter, and video-assisted thoracoscopic or robotic-assisted approaches are more favored [72]. A robotic-assisted approach allows for easier exposure of targets for ablation, avoidance of epicardial fat, and navigation of pericardial adhesions from prior inflammation or sternotomy [72,73]. Additionally, a robotic-assisted approach has greater precision in applying RF energy to the epicardial surface and without esophageal injury when performing PWI [72,73].

Choice of single-procedure HA, staged HA, or SA with selective endocardial assessment depends upon patient needs and logistical considerations. CONVERGE and HARTCAP show that single-procedure HA is safe and efficacious. A single-procedure HA avoids the need for a second operative date [61,62]. Only in certain cases is a single-procedure HA beneficial, such as a combined need for LAA closure with SA and CTI ablation in a patient who cannot tolerate two separate procedures [62]. A staged HA procedure benefits from interval lesion maturation and resolution of tissue edema and pericardial inflammation [64].

It is important to distinguish between planned staged HA and SA with selective endocardial assessment. SA with selective endocardial assessment necessitates interval post-operative re-evaluation after initial SA to determine patient need and timing for subsequent touch-up CA, and as a result facilitates a more individualized approach to ablation. Epicardial electroanatomic mapping, which would have previously only been performed endocardially, allows for touch-up ablation during initial SA, and it is likely that practice patterns for HA may shift to an initial SA with epicardial mapping, followed by a repeat CA and endocardial assessment in patients who have “failed” the SA [63,65]. As shown in the aforementioned trials, patients often require touch-up ablation on endocardial assessment, and evaluation for endocardial assessment remains important in patients after initial SA. Importantly, in patients who have undergone SA alone, recurrent AF could simply be the result of incomplete PVI at the time of the SA. Given that PVI remains the cornerstone of any ablation strategy, endocardial assessment of PVI and endocardial ablation as needed should be strongly considered.

A reasonable overall framework for ablation planning, especially in patients who fail initial CA, is initial CA followed by SA with subsequent selective endocardial assessment. Repeat CA is more appropriate in patients with significant operative risk limiting aggressive management with SA or HA. Factors that warrant further endocardial assessment after initial SA include clinical signs and symptoms on follow-up, such as symptomatic drug-refractory AF/AFL/AT post-ablation, or findings on epicardial mapping during SA such as residual PW voltage. Considerations in patient selection for each ablation strategy are summarized in Table 3.

### 4.4. Complications and Limitations of Hybrid Ablation

Contraindications of HA are similar to those of CA, such as the presence of left atrial thrombus. Prior cardiac surgery is a relative contraindication to HA when performed via a robotic approach [72,74]. Major adverse events reported in HA occurred in 7.8% of patients in the CONVERGE and CEASE-AF trials and 5% of patients in the HARTCAP-AF study, with complications including pericardial effusion, stroke, pericarditis, and phrenic nerve injury [61,62,64]. Reported complications of HA in these studies are listed in Table 4. Rates of major complications were not different between the HA and CA groups in CEASE-AF and HARTCAP-AF, which utilized a video-assisted thoracoscopic approach and a bipolar RF clamp [62,64]. In the CONVERGE trial, which utilized a unipolar RF catheter, HA had a significantly greater rate of major adverse events when compared to CA [61]. This was mainly due to three patients who had post-operative inflammatory pericardial effusions occurring 1–3 weeks later that resolved and led to no further complications [61]. Another possible explanation is that lesion creation with a unipolar RF catheter advanced through a subxiphoid approach is known to be more challenging, especially in patients with larger body habitus [72,75]. No AEFs, cardiac perforations, or deaths in patients undergoing HA were noted in these studies [61,62,64].

A recent retrospective analysis of the National Inpatient Sample (NIS) database identified 1925 patients who underwent HA from 2017 to 2022 [75]. While the data are largely observational and potentially subject to bias, the NIS database provides promising results from a substantial patient population and timescale. HA had significantly fewer major adverse cardiac events (MACEs), acute myocardial infarction, pericardial effusion and tamponade [75]. HA, however, had significantly greater operative complications, such as hemorrhage [75]. The rates of stroke were not different between patients receiving HA and CA [75].

Since HA inherently requires two procedures, operative time tends to be significantly longer than for CA alone [62,64]. Additionally, HA requires the expertise of both a cardiothoracic surgeon and a cardiac electrophysiologist who are both trained to collaborate in HA, limiting the feasibility of HA to medical centers with the appropriate staffing and resources.

## 5. Future Directions

Future directions include expanding upon long-term outcomes and procedural complications in HA as more patients undergo HA and more data are collected. The evidence supporting the safety and utility of HA involved use of a unipolar RF catheter or a bipolar RF clamp for the initial SA, with a subxiphoid, transdiaphragmatic, or video-assisted thoracoscopic approach [61,62,64]. Recent procedural developments such as a robotic-assisted approach in HA and epicardial electroanatomic mapping in SA have gained traction but have yet to be studied in large trials [18,63].

Future areas of study include outcomes with a robotic-assisted approach and/or epicardial mapping in both SA alone and HA. There is growing interest in epicardial PFA as well, with a need for continued study of the role of PFA in HA during both CA and SA. There remains notable variability in operative technique and energy selection in the current literature on HA, with a need for studies comparing different operative approaches and energies. The relationship between endocardial–epicardial dissociation and clinical outcomes in AF also warrants further exploration.

HA must be tested against alternative ablation strategies, such as SA alone or a sequential approach. Given the known benefits of a staged approach with a 6–18-week interval between procedures, a sequential approach of initial SA followed by the determination of whether a patient warrants subsequent endocardial CA may be a cost-effective strategy that potentially reduces operative risk for some patients by sparing a second procedure. Analyses of cost-effectiveness would be insightful in ascertaining the most economical procedure for common patient scenarios. Rhythm follow-up and measures of success also remain varied and nebulous among studies, and there remains a need to determine the optimal post-operative follow-up periods and appropriate modalities for rhythm monitoring after HA.

## 6. Conclusions

The rapidly growing prevalence of AF presents an ever-increasing burden on the healthcare system. Guidelines recommend early rhythm control in patients with AF. The outcomes for endocardial CA in patients with paroxysmal AF are encouraging. However, AF recurrence rates in patients with perAF and LS-perAF remain high following endocardial CA alone, despite the addition of lesion sets beyond PVI. Despite technological developments such as PFA, long-term success rates in these patient populations remain suboptimal. Ensuring lesion transmurality remains critical. Endocardial–epicardial dissociation may contribute to suboptimal outcomes, although its specific relationship with clinical outcomes post-ablation warrants further study. Initial small-trial data suggest that HA seems safe and efficacious in carefully selected patients with perAF and LS-perAF, particularly those who have failed prior CA.

## Figures and Tables

**Table 1:** Study Design.

Study	Population	Baseline Antiarrhythmic Drugs and Prior Ablations	Procedural Approach and Lesion Set	Follow-Up Rhythm Monitoring
CONVERGE (2020) [61]	- 102 patients in HA Group. - 51 patients in CA Group. - Of all patients, 58% with perAF and 42% with LS-perAF.	- 84% in HA group on Class I/III AADs. - 80% in CA group on Class I/III AADs. - All patients with no prior ablations	- Single-procedure HA with SA and CA in series. - SA via pericardioscopy. 65.7% with a transdiaphragmatic approach and 34.3% with a subxiphoid approach. - Epicardial lesion sets created with AtriCure EPi-Sense System, a unipolar RF catheter. - Patients received epicardial PWI followed by endocardial PVI.	- 3-month blanking period - 24 h Holter monitor at 6 and 12 months - 7-day Holter monitor at 18-months or longer.
HARTCAP-AF (2023) [62]	- 19 patients in HA Group. - 22 patients in CA Group. - In the HA group, 90% with perAF and 10% with LS-perAF. - In the CA group, 91% with perAF and 9% with LS-perAF.	- 53% in HA group on Class I/III AADs. - 36% in CA group on Class I/III AADs. - All patients with no prior ablations	- Single-procedure HA with SA and CA in series. - For initial SA, 74% with left-sided unilateral video-assisted thoracoscopic approach. 26% with a bilateral approach, if unable to ablate right PVs. - Epicardial lesion sets created with AtriCure Synergy System and a bipolar RF clamp. - Patients received epicardial PVI, PWI with a box lesion, and LAA isolation with AtriClip clipping device or Lariat closure device.	- 3-month blanking period - 12-lead ECG and 24 h Holter monitor at 3 and 6 months. - 7-day Holter monitor at 12 months or longer.
CEASE-AF (2023) [64]	- 102 patients in HA group. - 52 patients in CA group. - In the HA group, 79.4% with perAF and 20.6% with LS-perAF. - In the CA group, 82.7% with perAF and 17.3% with LS-perAF.	- 53.9% in HA group on Class I/III AADs. - 57.7% in CA group on Class I/III AADs. - All patients with no prior ablations	- Staged HA procedure with initial SA followed by CA 91–180 days later. - For initial SA, bilateral video-assisted thoracoscopic approach. - Epicardial lesion sets created with AtriCure Synergy System, a bipolar RF clamp. - Patients received epicardial PVI and PWI with a box lesion. 92% of patients received LAA isolation with the AtriClip clipping device.	- 3-month blanking period - 48 h Holter monitor and 12-lead ECG

**Table 2:** Study Outcomes.

Study	HA Group—Freedom from AF/AFL/AT	CA Group—Freedom from AF/AFL/AT	*p*-Value
CONVERGE (2020) [61]	67.7% at 12 months off new or increased AADs	50.0% at 12 months off new or increased AADs	0.025
	60.5% at 18 months off new or increased AADs in a LS-perAF subgroup	25.9% at 18 months off new or increased AADs in a LS-perAF subgroup	<0.01
HARTCAP-AF (2023) [62]	89% at 12 months off AADs	41% at 12 months off AADs	<0.01
	57% at 36 months off AADs	22.7% at 36 months off AADs	<0.01
CEASE-AF (2023) [64]	71.6% at 12 months off new or increased AADs	39.2% at 12 months off new or increased AADs	<0.01
	66.3% at 24 months off new or increased AADs	33.3% at 24 months off new or increased AADs	<0.01

**Table 3:** Considerations in Selecting an Ablation Strategy.

Ablation Strategy	Considerations for Selection
Catheter ablation	Appropriate initial procedure in patients with perAF or LS-perAF. May be considered for redo procedure in patients with significant operative risk limiting SA. Allows for concomitant CTI ablation for AFL and ablation of non-PV triggers of AF along with other inducible arrhythmias.
Surgical ablation	Appropriate in patients who failed prior CA(s) [33]. Appropriate for low operative risk patients with risk factors (age, LA size, AF duration) predicting CA failure [23,24,26,32]. Appropriate for patients undergoing cardiac surgery. Greater operative risk when compared with CA [71]. Operative procedure allows for LAA clipping or closure [62,64].
Surgical ablation with selective endocardial assessment	Includes all the aforementioned benefits of SA. Allows for flexibility in determining whether patient requires subsequent endocardial assessment and ablation. Intraoperative epicardial mapping may reduce the need for any subsequent endocardial assessment [63,65].
Staged hybrid ablation	Includes all the aforementioned benefits of SA. Compared to single-procedure HA, allows for lesion maturation and reduction of tissue edema and pericardial inflammation, improving accuracy of subsequent endocardial assessment and touch-up ablation [64].
Single-procedure hybrid ablation	Includes all the aforementioned benefits of SA. Most appropriate for patients who may not be able to tolerate a staged procedure but requires epicardial and endocardial management; for example, the need for LAA closure and CTI ablation for AFL Necessitates systemic anticoagulation in a patient with fresh surgical incisions.

**Table 4:** Study Adverse Events.

Study	Rate of Major Adverse Events	Adverse Events
CONVERGE (2020) [61]	8 (7.8%)	- 3 pericardial effusions - 1 phrenic nerve injury - 1 transient ischemic attack - 1 stroke - 2 episodes of bleeding - No cardiac perforations, atrioesophageal fistula, or death.
HARTCAP-AF (2023) [62]	1 (5%)	- 1 case of pericarditis - No cardiac perforations, atrioesophageal fistula, or death.
CEASE-AF (2023) [64]	8 (7.8%)	- 1 case of stroke and myocardial infarction - 1 case of pericarditis - 1 episode of bleeding - 1 vascular access complication - 1 pneumothorax - 1 pneumonia - 1 permanent pacemaker implantation - 1 episode of aspiration - No cardiac perforations, atrioesophageal fistula, or death.

## Data Availability

No new data were created or analyzed in this study. Data sharing is not applicable to this article.

## Abbreviations

The following abbreviations are used in this manuscript:

AF	Atrial Fibrillation
perAF	Persistent Atrial Fibrillation
LS-perAF	Long-Standing Persistent Atrial Fibrillation
RF	Radiofrequency
CB	Cryoballoon
PFA	Pulsed Field Ablation
IRE	Irreversible Electroporation
AEF	Atrioesophageal Fistula
PVI	Pulmonary Vein Isolation
PV	Pulmonary Vein
PW	Posterior Wall
PWI	Posterior Wall Isolation
VOM	Vein of Marshall
CFAE	Complex Fractionated Atrial Electrograms
CTI	Cavotricuspid Isthmus
HA	Hybrid Ablation
CA	Catheter Ablation
TEE	Transesophageal Echocardiography
LAA	Left Atrial Appendage
AFL	Atrial Flutter
AT	Atrial Tachycardia
AAD	Antiarrhythmic Drug
MACE	Major Adverse Cardiac Event
